# Epidemiology of dengue and other arboviruses in a cohort of school children and their families in Yucatan, Mexico: Baseline and first year follow-up

**DOI:** 10.1371/journal.pntd.0006847

**Published:** 2018-11-21

**Authors:** Diana Patricia Rojas, Gloria Abigail Barrera-Fuentes, Norma Pavia-Ruz, Mariel Salgado-Rodriguez, Azael Che-Mendoza, Pablo Manrique-Saide, Gonzalo M. Vazquez-Prokopec, M. Elizabeth Halloran, Ira M. Longini, Hector Gomez-Dantes

**Affiliations:** 1 Department of Biostatistics, University of Florida, Gainesville, FL, USA; 2 Center for Inference and Dynamics of Infectious Diseases, Seattle, WA, USA; 3 Centro de Investigaciones Regionales Dr. Hideyo Noguchi, Universidad Autonoma de Yucatan, Merida, Yucatan, Mexico; 4 Campus de Ciencias Biologicas y Agropecuarias, Universidad Autonoma de Yucatan, Merida, Yucatan, Mexico; 5 Department of Environmental Sciences, Emory University, Atlanta, GA, USA; 6 Vaccine and Infectious Disease Division, Fred Hutchinson Cancer Research Center, Seattle, WA, USA; 7 Department of Biostatistics, University of Washington, Seattle, WA, USA; 8 Center for Health Systems Research, National Institute of Public Health, Cuernavaca, Morelos, Mexico; University of California, Berkeley, UNITED STATES

## Abstract

Dengue is the most prevalent mosquito-borne viral disease of humans and is caused by the four serotypes of dengue virus. To estimate the incidence of dengue and other arboviruses, we analyzed the baseline and first year follow-up of a prospective school-based cohort study and their families in three cities in the state of Yucatan, Mexico. Through enhanced surveillance activities, acute febrile illnesses in the participants were detected and yearly blood samples were collected to evaluate dengue infection incidence. A Cox model was fitted to identify hazard ratios of arboviral infections in the first year of follow-up of the cohort. The incidence of dengue symptomatic infections observed during the first year of follow-up (2015–2016) was 3.5 cases per 1,000 person-years (95% CI: 1.9, 5.9). The incidence of dengue infections was 33.9 infections per 1,000 person-years (95% CI: 31.7, 48.0). The majority of dengue infections and seroconversions were observed in the younger age groups (≤ 14 years old). Other arboviruses were circulating in the state of Yucatan during the study period. The incidence of symptomatic chikungunya infections was 8.6 per 1,000 person-years (95% CI: 5.8, 12.3) and the incidence of symptomatic Zika infections was 2.3 per 1,000 person-years (95% CI: 0.9, 4.5). Our model shows that having a dengue infection during the first year of follow-up was significantly associated with being female, living in Ticul or Progreso, and being dengue naïve at baseline. Age was not significantly associated with the outcome, it was confounded by prior immunity to dengue that increases with age. This is the first report of a cohort in Latin America that provides incidence estimates of the three arboviruses co-circulating in all age groups. This study provides important information for understanding the epidemiology of dengue and other arboviruses and better informing public health policies.

## Author summary

Dengue is a major public health problem worldwide. The burden of dengue in Latin America is been increasing in the last years and Mexico is one of the countries with the highest burden in this region. To better understand the transmission of dengue in Mexico we established a school-based prospective cohort study of dengue virus infection in children and their families in three cities in the state of Yucatan, Mexico. In this article we show that around 70% of the cohort population has prior immunity to dengue. Also that the rate of dengue cases varied among cities and we also confirm the circulation of chikungunya and Zika virus in the cohort participants during the first year of follow-up. We found evidence that having prior dengue immunity might protect again Zika symptomatic infections. The results of this study will be useful for the scientific community and policy makers from Mexico and other countries in Latin America to properly plan and evaluate control strategies and interventions like vector control and vaccines.

## Introduction

Over the past 40 years, dengue virus incidence in human population has increased 30-fold and the geographic range of the virus expanding to new countries as increasing urbanization, global human travel and urban to rural migration enable opportunities for transmission [1, 2] Currently more than 40% of the human population worldwide is at risk of dengue infections, with approximately 390 million infections estimated to occur globally each year, of which 96 million have clinical manifestations of the virus [3].

Dengue virus (DENV) has four serotypes, each of them can be responsible for dengue epidemics and can also be associated with severe disease depending on the sequence and time between infections, among other factors [4–6]. The clinical spectrum of dengue ranges from asymptomatic infections to life-threatening severe disease; approximately 50% to 90% of dengue infections are asymptomatic [7, 8]. The probability of symptomatic dengue due to dengue virus infection is altered by the previous dengue immune status [9]. Currently there is no specific treatment for dengue infection or clinical predictors to prevent severe disease [10].

Surveillance systems based on the monitoring and notification of dengue symptomatic cases have low sensitivity and rarely detect low or sporadic transmission [11, 12]. The proportion of dengue asymptomatic infections varies widely by populations, geographical areas, and over different epidemiological periods [13]. Underreporting of dengue cases to national surveillance systems hinders accurate local, regional and global estimation of disease burden [14]. Necessary steps to better understand dengue transmission in at-risk populations include strengthening passive surveillance systems by incorporating active surveillance methods (e.g., house-to-house visits, school absenteeism or self-identification of fever episodes) and improving the detection of inapparent infections to rectify the underreporting of unspecified febrile dengue infections.

Vector control remains the core strategy for the prevention of dengue [15, 16]. However, current approaches that only temporarily affect mosquito populations have not been proven to be sufficient to prevent dengue transmission [17]. Effective and sustainable dengue prevention and control requires innovation, by integrating and making optimal use of the most effective control tools available like integrated vector management, and also the introduction of new tools e.g. vaccines and other novel vector control technologies, especially in growing and complex urban environments [18, 19].

Currently five vaccine candidates are in clinical stages of development [20]. Only one vaccine has completed two Phase III trials and it is being licensed for introduction in several countries [21–23]. This vaccine has an estimated efficacy of 64.7% and 56.5% in the Latin American and Southeast Asian trials, respectively and the efficacy was significantly higher in participants with pre-existing dengue neutralizing antibodies compared to those who were seronegative [21, 22, 24].

The availability of a licensed vaccine poses different challenges to current dengue control programs since it is not expected to vaccinate all the susceptibles and at-risk populations or even provide complete vaccine coverage in target groups. Vaccine introduction will therefore be a gradual process. In each of these populations several questions need to be addressed regarding the clinical spectrum and transmission risks in order to target vaccination to provide the most benefit [25].

Thus, measuring the burden of disease attributable to dengue infection is imperative for understanding the potential impact of dengue control interventions and feasibility for vaccine introduction and evaluation. There are limited number of established longitudinal cohort studies characterizing dengue infection in Latin America. The aim of this study was to estimate age-specific attack rates, the proportion of inapparent infections, incidence rate ratios and assessing the covariates associated with arboviral infections and symptomatic disease in a prospective dengue cohort established in Yucatan, Mexico.

## Methods

### Settings

The state of Yucatan is located in the southeast peninsula of Mexico, bordering the Gulf of Mexico and the Caribbean Sea. As described elsewhere, the cities of Merida, Progreso and Ticul were selected as three settings with different epidemiological dynamics (low, medium, high dengue transmission) (Table 1), based on their history of dengue outbreaks in the past and the co-circulation of more than two serotypes in recent years [26]. Incidence rates of suspected and confirmed cases from 1979 to 2013 were estimated for each city. As these three cities also have different dengue incidence rates, this allowed us to explore possible differences in dengue transmission scenarios [27].

**Table 1:** Characteristics of the study settings of the cohort in Yucatan, Mexico.

	Merida	Progreso	Ticul
Population (inhabitants)	892,363	59,122	40,161
Number of households	257,826	16,020	9,808
Average yearly temperature (°C)	25.9	25.4	26.4
Precipitation (mm)	1,050	466	1,229
Historical dengue risk	High risk	Medium risk	Low risk

Merida, the capital city, is the largest urban center in the region with 830,732 inhabitants (2016). The weather is warm and humid, the mean annual temperature is 25.9°C (19.5 to 33.6) and annual precipitation is 1050 (mm). The rainy season is from June to October. Progreso is the main seaport in the state, 32 km away from Merida, with 54,000 inhabitants. This town has similar weather conditions and is a weekend and holiday resort (July-August) for people living in Merida, as well as a national and international tourist resort. Ticul is a town located 82 km south of Merida with 34,000 inhabitants whose main economic activity is dedicated to shoe manufacturing [28].

### Selection of risk areas

The primary aim of this study was to characterize the local epidemiology and transmission dynamics of dengue in the state of Yucatan, using a school-based, prospective cohort in the different transmission district by city, three criteria were adopted: 1) Proportion of epidemiological weeks with reported symptomatic dengue cases compared to epidemiological weeks with no reported cases; 2) Duration of the dengue epidemic waves as a proxy of persistent dengue transmission; 3) Intensity of transmission measured as the incidence rate during the dengue season.

The school-based study is defined by a random selection of five extensive geographic areas (districts) with different dengue transmission risks (high, medium or low). The data obtained from the epidemiological surveillance system identified those areas where dengue has been historically higher and persistent and where the transmission risk is defined as high, medium and low. The sample includes low-risk areas in Merida and Progreso, medium-risk areas in Merida and Ticul, and a high-risk area in Merida. The sample size was estimated for the school-children from each transmission risk by city. The sample size was 150 school children from each transmission risk by city and then expanded to their families.

Each area of transmission included several primary public schools from which the cohort of children from first to third grade was randomly selected. The randomization was done using stratified random sampling. First, we stratified the transmission risk of the neighborhoods on the three cities (Merida, Progreso and Ticul). These included two low-risk areas (one in the north of Merida and one in Progreso); two medium risk areas (one urban area in central Merida and one in Ticul); and one high-risk urban area in the south of Merida. The risk was defined based on historical epidemiological features: 1) the historical dengue cases reported to the epidemiological surveillance system; 2) the percent of cases reported every year in each setting; and 3) the continuous transmission during 6 to 8 or more weeks every year. Second, after selecting some areas randomly based on the transmission risk, the field team asked for the list of all the schools located on the randomized transmission settings and a simple random sampling procedure was done to select the schools. The next step was done with the list of the students from 1st to 4th grade from each school that were also randomized.

### Recruitment and enrollment

Children were enrolled at the beginning of the academic year from first through third grade (6 to 8 years old) and were eligible to remain in the study until they graduated from sixth grade (12 years-old). The only exclusion criterion was intent to move outside of the study area during the twelve months following enrollment. The students entering elementary school and those in second and third grade were selected and included in the cohort study with written consent granted by their parents. Each group of enrolled children and their respective families were followed-up during their education track for the period of the study. Families were defined for this study as people living in the same household (with minimum of five nights sleping in the household) but not necessary that belong to the same family (blood relation).

The strategy of establishing and maintaining a cohort of individuals and their families in a long-term project requires ongoing effort. Success of such projects requires sharing interim results, advances in the project, and deep understanding of the community-level protective measures against dengue epidemics. Therefore, each site had a team of community specialists consisting of local physicians, nurses, laboratory technicians, microbiologists, and anthropologists who gathered information and organized activities directed to sensitize and educate cohort members and their families about dengue epidemiology and control.

### Follow-up visits

We obtained demographic information and blood samples from the entire study population at baseline and an annual blood sample. The study participants and their families were followed from January 2015 through October 2016 at 12-month intervals for serological evidence of dengue infection after the dengue season every year. Blood samples were taken during the surveillance period each year (January-July) right after the typical dengue transmission season. Participants were considered lost to follow-up if a full year had passed since their previous blood draw, despite repeated attempts to locate the participant, or if there was a verifiable reason for dropping them from the study (e.g., direct request from the participant, movement from the study area, or death).

During the follow-up period the infection rates in different age groups were measured as well as the proportion of inapparent infections or underreporting of febrile cases during the dengue season. The prospective follow-up of the school cohort and their families also provided information for the estimation of age group hazards of infection for those represented in this sample.

#### Enhanced surveillance

The enhanced surveillance was used to identify dengue acute febrile cases in the cohort through absentee surveillance at school and its surrounding area, and also from the Mexican national surveillance system.

In addition to weekly school visits to investigate school absenteeism there was continuous active surveillance for febrile cases in all the cohort participants using mobile applications and a dengue toll free phone number (1-800). The mobile application was used by the staff of the study to collect the socio-demographic and geographic information for each participant at enrollment and during the periodic visits to the households. The toll free phone was a 24/7 phone line ready to receive reports of febrile cases that met the case definition of dengue for the protocol. Every call received on this toll free phone that suggested a dengue case triggered a visit to the family by a physician from the study who activated the procedures described on the study protocol to detect dengue infections. Also the families were advise to report hospitalizations and any changes on the address of the participants of the cohort.

The cohort participants were followed closely for all febrile illnesses, and children and their relatives who also had fever were screened clinically and by laboratory testing, for signs and symptoms of dengue. The surveillance system identified suspected or probable dengue cases that were clinically and serologically studied to confirm dengue infection. During school vacation (July-August and December-March), children were visited or contacted weekly at their homes. During the annual follow-up visits, the field team collected blood samples from all the participants of the study who consented to give the blood sample in order to detect inapparent infections in the cohort.

A person was considered to have an inapparent (subclinical) dengue infection when their serum sample tested positive for IgM antibody to any dengue serotype without an accompanying febrile illness or school-absence identified during the active surveillance period or if the cohort participant seroconverted in the annual follow-up samples. Clinical definitions of serologically or virologically confirmed dengue infection were based on evidence of acute dengue infection, as described below.

#### Case definitions

Symptomatic dengue infection was classified as symptomatic non-hospitalized or symptomatic hospitalized, based on admission into the hospital as decided by the treating physician. Hospitalized symptomatic cases were further defined as either dengue or severe dengue using the 2009 guidelines from the World Health Organization [1].

*Dengue case definition*. Dengue cases were classified according to the 2009 WHO Case Classification [1].

*Dengue without warning signs*. Dengue without warning signs is defined by acute fever (≥ 38.5°) and two or more of the following symptoms: headache, myalgia, arthralgia, retroorbital pain, rash, hemorrhagic manifestations or leukopenia [1].

*Dengue with warning signs*. Dengue fever with any of the following warning signs: abdominal pain, persistent vomiting, fluid accumulation, lethargy, mucosal bleeding, fluid accumulation, liver enlargement, or increasing hematocrit with decreasing platelets [1].

*Severe dengue*. Dengue fever with any of the following: severe bleeding, severe plasma leakage leading to shock or fluid accumulation with respiratory distress, or organ failure or involvement as evidenced by liver enzymes ≥ 1, 000, impaired consciousness, failure of the heart or other organs [1].

#### Laboratory criteria for confirmation for dengue

*Acute dengue case*. A symptomatic participant with less than 7 days of onset of symptoms who tested positive for dengue evidenced by: 1) detection of dengue NS1 antigen, 2) detection of IgM antibodies to dengue virus by ELISA, 3) detection of IgG capture antibodies to dengue virus, or 4) detection of DENV RNA by Trioplex Real-time RT-PCR [29–34].

*Inapparent dengue infection*. A participant whose paired annual serum samples demonstrated seroconversion by four-fold increase on IgG antibodies and who did not experience a symptomatic dengue infection during the time of follow-up [29, 35–37].

*Primary dengue infections*. A dengue infection is classified as a primary DENV infection if seroconversion was observed [38]. First DENV infections were identified by counting the number of the infections documented in a participant who entered the cohort dengue-naïve, and whose paired annual sample were positive for anti-DENV IgG antibodies [29, 37, 38].

#### Dengue prior exposure

*Dengue-naïve*. A participant who did not have detectable anti-DENV antibody at enrollment when their sample was tested using a Dengue IgG Indirect ELISA.

*Dengue non-naïve*. A participant who had detectable anti-DENV antibody at enrollment when their sample was tested using a Dengue IgG Indirect ELISA at enrollment [29, 37, 38].

### Chikungunya case definition

#### Acute chikungunya symptomatic infection

A participant with the presence of all these signs and symptoms: abrupt onset of fever ≥ 38.5° accompanied by joint pain, muscle pain, headache, nausea, fatigue and rash [39].

#### Laboratory criteria for confirmation for chikungunya

A symptomatic participant who tested positive for chikungunya evidenced by: 1) detection of CHIKV RNA by RT-PCR or Real-time RT-PCR, or 2) enzyme-linked immunosorbent assays (ELISA) confirmed anti-chikungunya IgM antibodies [39].

### Zika case definition

#### Zika virus disease

Patient with rash and two or more of the following signs or symptoms: fever, usually ≥ 38.0°, conjunctivitis (non-purulent/hyperemic), arthralgia or myalgia [40, 41].

#### Laboratory criteria for Zika confirmation

Participant who met the criteria for a suspected case AND had laboratory confirmation of Zika virus RNA in serum or urine by Real-time RT-PCR [42]. For ZIKV diagnosis, serological tests like IgM ELISA were not used in this study to confirm ZIKV disease due to the cross-reactivity of the tests available with other flaviviruses circulating in the region like DENV [43, 44].

### Ethics statement

This study was conducted as a collaboration between the Ministry of Health of Yucatan, Mexico and the Center for Inference and Dynamics of Infectious Diseases in Seattle, WA, USA. This study was approved by the Institutional Review Boards at Fred Hutchinson Cancer Research Center, Seattle, WA, USA and the General Hospital Agustin O’Horan, Health Services of Yucatan, Mexico. Written consent was obtained from all adult participants (>18 years old) after providing them with a detailed explanation of the study and procedures. Parents/guardians of all child participants (≤ 18 years old) were asked to provide written consent on their behalf. The analysis using de-identified data was approved at Fred Hutchinson Cancer Research Center.

### Statistical analysis

The age-specific baseline seroprevalence for dengue virus was estimated for the entire cohort. For the first year of follow-up, the follow-up time was estimated as the time between enrollment and the end of the reported study period (August 2016), or withdrawal from the study. For those who were lost to follow-up, person-years were calculated as the time between enrollment and the last contact with study personnel, plus one-half the time between the last contact and the date recorded as lost to follow-up.

The analysis of dengue infections was limited to those participants who completed the year and contributed a blood sample at the beginning and the end of the year. In order to provide estimates of dengue infection incidence, since the exact timing of the dengue infection could not always be ascertained, persons who experienced a dengue infection in the first year of follow-up contributed person-time for that entire year. For the analysis of dengue infections, the age of the participant was defined as the age when their annual sample was collected. The crude incidence per 1,000 person-years was 1,000 times the number of dengue infections divided by the number of person-years in the dataset as published elsewhere [29, 35, 45].

The incidence rate ratio was also estimated for each arboviral infection comparing dengue non-naïve and dengue naïves [46–48]. The hazard ratios (HRs) and 95% confidence intervals (95% CIs) for dengue and other arboviral infections were estimated using a Cox proportional hazards model and adjusting for potential confounders such as age, gender, city, and prior exposure to dengue [48, 49]. All the statistical analyses were done in R, version 3.2.1 [50].

## Results

### Baseline characteristics of the participants

**Fig 1:**
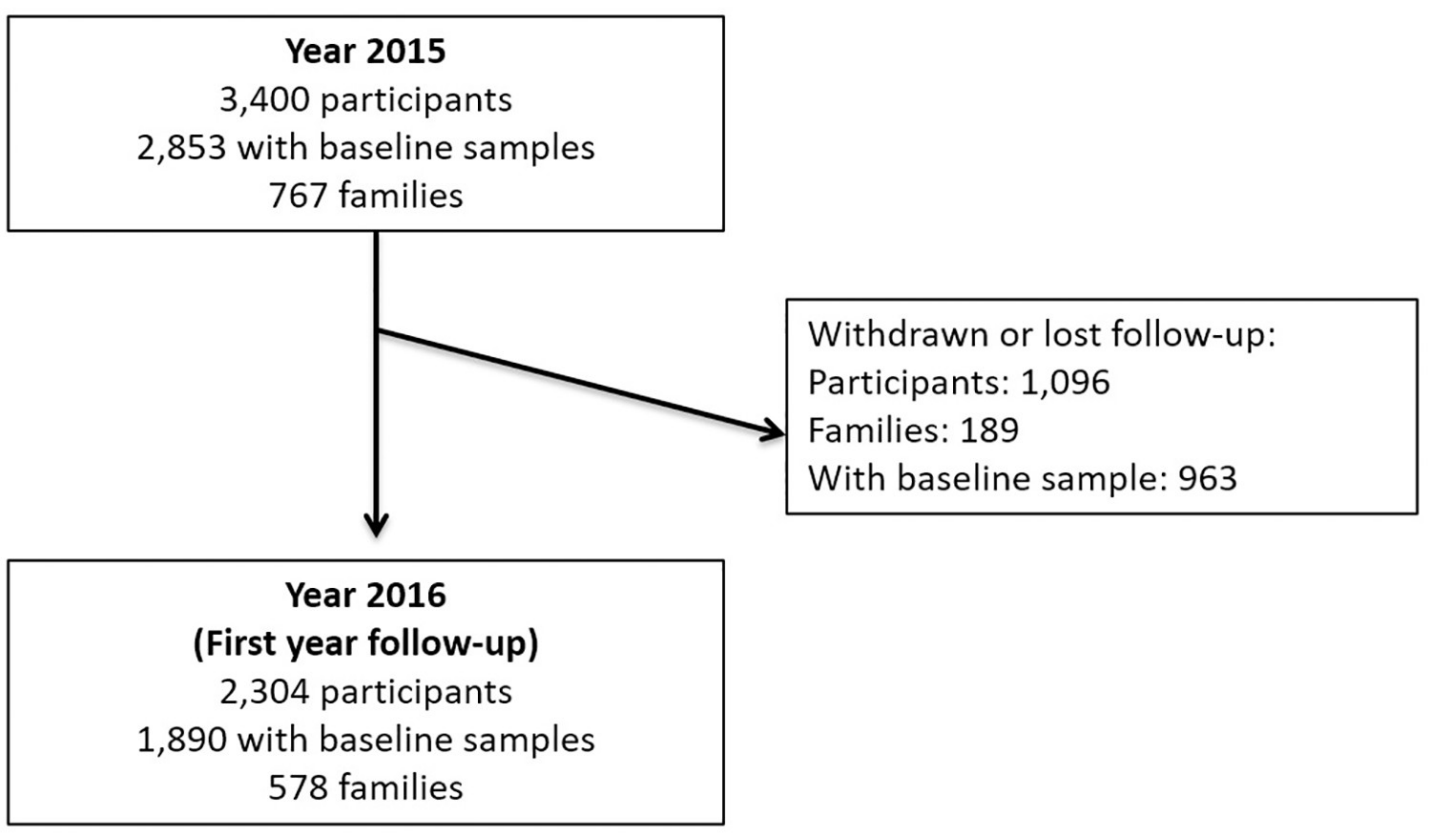
Flowchart of participants in the dengue cohort, Yucatan, Mexico.

A total of 767 families encompassing 3,400 participants were enrolled from January to June, 2015 across the three sites in Yucatan. The majority of the families and participants were from Merida (2,021 (59.4%), followed by Ticul (738 (21.7%) and Progreso (641(18.9%) (Fig 1). Among the participants 1,869 (55.0%) were females, 1,463 (43.0%) were 14 years old or younger, and 2,970 (87.4%) of them were born in the state of Yucatan (Table 2). These participants contributed with 3430.87 person-years for the first year of follow-up. The mean participation time is 1.01 years (368.31 days) per participant (range 0.04–1.94).

**Table 2:** Demographic characteristics of the enrolled cohort population from Yucatan, Mexico in 2015 (N = 3,400).

Variable	Merida (N = 2,021)	Progreso (N = 641)	Ticul (N = 738)	Total
Age (Years)				
<8	593 (29.3%)	188 (29.3%)	198 (26.8%)	979 (28.8%)
9–14	289 (14.3%)	86 (13.4%)	109 (14.8%)	484 (14.2%)
15–19	85 (4.2%)	33 (5.2%)	40 (5.4%)	158 (4.7%)
20–49	842 (41.7%)	277 (43.2%)	337 (45.7%)	1456 (42.8%)
>50	212 (10.5%)	57 (8.9%)	54 (7.3%)	323 (9.5%)
Gender				
Male	917 (45.4%)	286 (44.6%)	328 (44.4%)	1531(45.0%)
Female	1104 (54.6%)	355 (55.4%)	410 (55.6%)	1869 (55.0%)
Number of families	463 (60.3%)	153 (20.0%)	151 (19.7%)	767 (100%)
Average number of people per family (Range)	4.36 (2,11)	4.18 (2,11)	4.88 (2,16)	4.43
Born in Yucatan				
Yes	1775 (87.8%)	550 (85.8%)	645 (87.4%)	2970 (87.4%)
No	129 (6.4%)	52 (8.1%)	46 (6.2%)	227 (6.7%)
No information	117 (5.8%)	39 (6.1%)	47 (6.4%)	203 (6.0%)
Withdrawn (No follow-up)				
Yes	778 (38.5%)	123 (19.2%)	195 (26.4%)	1096 (32.2%)
No	1243 (61.5%)	518 (80.8%)	543 (73.6%)	2304 (67.8%)

A total of 1,096 (32.2%) participants did not complete the first year of follow-up. A total of 320 (29.3%) of the participants were classified as lost to follow-up (the team lost track of the participant and the family), 210 (19.2%) moved out of the state of Yucatan and 564 (52.0%) asked for voluntary withdrawal from the study (Fig 1).

### Dengue baseline seroprevalence

**Fig 2:**
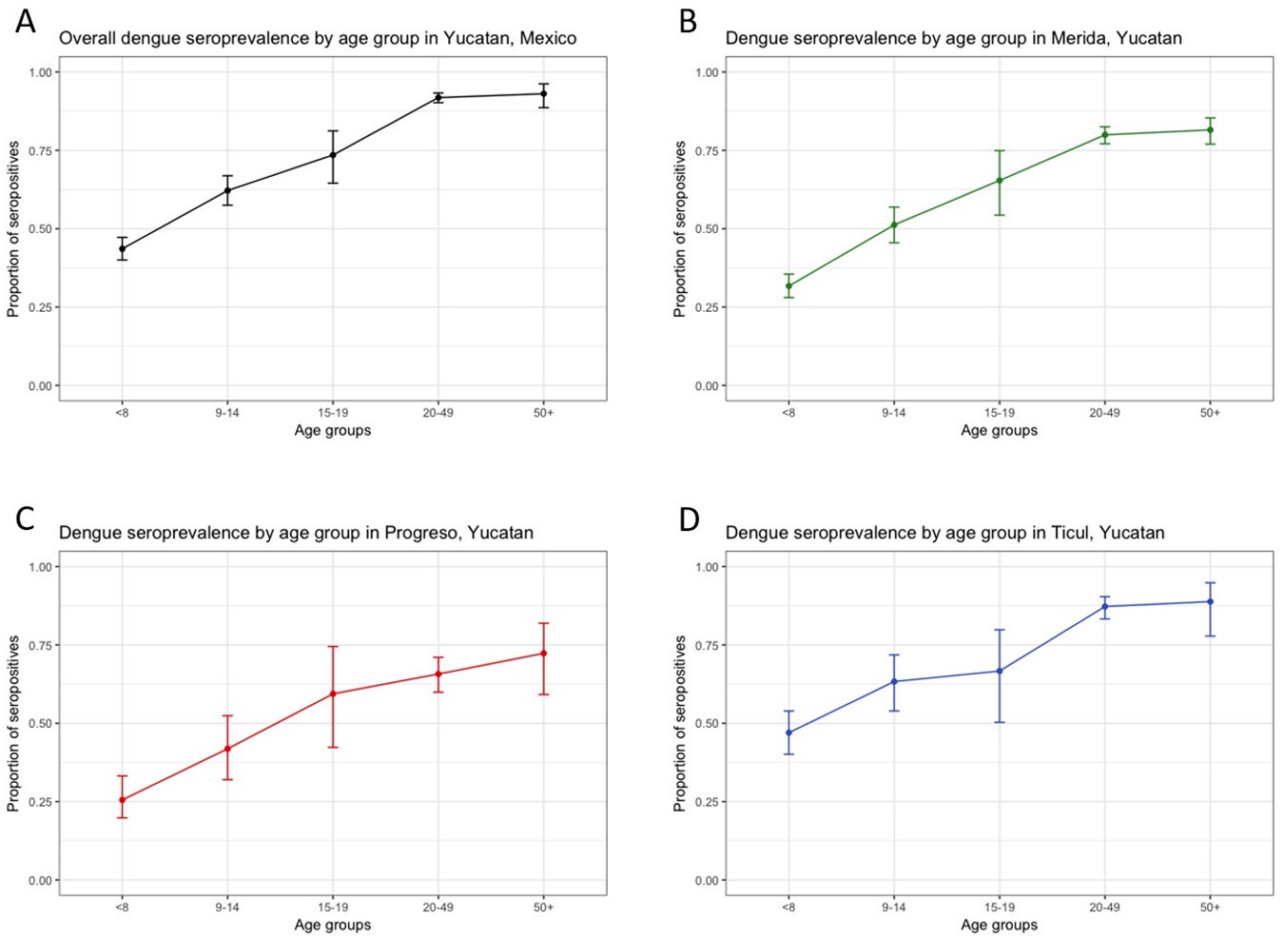
Age-specific baseline dengue seroprevalence in the cohort, Yucatan, Mexico. A. Overall dengue seroprevalence of the cohort by age. B. Dengue seroprevalence of the cohort in Merida by age. C. Dengue seroprevalence of the cohort in Progreso by age. D. Dengue seroprevalence of the cohort in Ticul by age.

The baseline seroprevalence was estimated using samples from 2,853 participants who authorized a blood sample collection at enrollment. The overall baseline dengue seroprevalence in the cohort was 70.28% (95% CI 68.6%–72%) indicating that most of the population had already been exposed to at least one dengue infection (Fig 2).

The cohort from Ticul had the highest seroprevalence (81.1%), followed by Merida (70.2%) and Progreso (57.9%). The differences in seroprevalence estimates by city were statistically significant (p<0.001). Dengue seroprevalence increased with age, and females in the cohort had significantly higher seroprevalence estimates compared with males (p = 0.04) (Table 3).

**Table 3:** Baseline dengue seroprevalence of enrolled cohort of participants from Yucatan, Mexico, in 2015, by city, age and gender (n = 2,853).

Baseline exposure	Dengue-naïve (n = 829)	Dengue non-naïve (n = 2005)	Indeterminate (n = 19)	Total
City				
Merida	490 (29.2%)	1178 (70.2%)	10 (0.6%)	1678
Progreso	226 (41.7%)	313 (57.9%)	2 (0.4%)	541
Ticul	113 (17.8%)	514 (81.1%)	7 (1.1%)	634
Age (Years)				
≤ 8	484 (59.0%)	329 (40.1%)	8 (1.0%)	821
9–14	184 (41.7%)	253 (57.4%)	4 (0.9%)	441
15–19	37 (29.8%)	86 (69.4%)	1 (0.8%)	124
20–49	109 (8.6%)	1149 (90.9%)	6 (0.5%)	1264
≥ 50	15 (7.4%)	188 (92.6%)	0 (0%)	203
Gender				
Male	416 (34.0%)	792 (64.8%)	14 (1.2%)	1222
Female	413 (25.3%)	1213 (74.4%)	5 (0.3%)	1631

### Suspected symptomatic arboviral cases

In the time period after enrollment and the collection of the baseline blood sample to the first annual follow-up of the cohort, 199 suspected arbovirus infection cases or undifferentiated febrile illnesses were identified in the study population of 3,400. The most common clinical diagnosis was undifferentiated fever (105 cases (52.76%)), followed by 76 (38.19%) suspected dengue cases, 17 (8.54%) suspected chikungunya cases and one suspected case of Zika. (Table 4). The dengue incidence rate for suspected cases was 21.86 per 1,000 person-years. From the 199 samples tested, 148 (74.4%) were negative for dengue, chikungunya y Zika.

**Table 4:** Arbovirus suspected cases by etiology in cohort participants in Yucatan, Mexico (N = 199).

	Dengue	Zika	Chikungunya	Indeterminate	Negative	Total
Probable symptomatic cases						
Dengue	7 (58.3%)	1 (12.5%)	11 (36.7%)	1 (100%)	56 (37.8%)	76 (38.2%)
Chikungunya	1 (8.3%)	6 (75%)	0	0	10 (6.8%)	17 (8.5%)
Zika	1 (8.3%)	0	0	0	0	1 (0.5%)
Undifferentiated fever	3 (25%)	1 (12.5%)	19 (63.3%)	0	82 (55.4%)	105 (55.4%)
Total confirmed	12	8	30	1	148	199

All the consultations were in the outpatient clinic. The most common symptoms presented by the cohort population were: fever, headache, myalgia, arthralgia, rash and conjunctivitis. No significant differences in symptoms were found among all the arboviral clinical diagnoses (p = 0.560). No severe symptoms were identified in the study population.

As the symptoms were non-specific across the different arboviral infections, we estimated the incidence of arbovirus suspected cases to be 58.02 per 1,000 person years.

### Confirmed arboviral symptomatic infections

A total of 199 participants with suspected arbovirus symptomatic infections or undifferentiated febrile illnesses were identified in the 2935 participants of the cohort. These participants contributed with 3236.48 person- years in the first annual follow-up. Among the symptomatic arboviral infections 12 (6.0%) were laboratory-confirmed as dengue-positive, 30 (15.1%) as chikungunya-positive, 8 (4.0%) as Zika-positive, and 148 (74.4%) were classified as fever of unknown origin (Table 4). The overall incidence rate of arbovirus confirmed symptomatic infections was 14.57 per 1,000 person-years (95% CI: 10.82, 19.21).

The incidence rate of confirmed symptomatic dengue was 3.45 cases per 1,000 person-years (95% CI: 1.87, 5.86). In the first year of the study, 12 symptomatic acute dengue cases were serotyped; DENV1 (52%) was the most isolated serotype, followed by serotype DENV4 (33.3%) and DENV2 (16.7%). DENV3 was not isolated during the analyzed period. No hospitalizations or deaths resulting from symptomatic dengue infection were reported during the first year of the cohort. The highest incidence of dengue was observed in the participants in the following groups: participants that were 15-19 years of age (IR: 6.88 per 1,000 person-years), females (IR: 6.3 per 1,000 person-years), and participants from Merida (IR: 4.2 per 1,000 person-years) (Table 5). The incidence rate of confirmed symptomatic chikungunya was 8.62 cases per 1,000 person-years (95% CI: 5.81, 12.30). The highest incidence of chikungunya was observed in the participants in the group 50 years old or older, females and in Ticul. (Table 5). The incidence rate of confirmed symptomatic Zika was 2.33 cases per 1,000 person-years (95% CI: 0.99, 4.53). The highest incidence rate estimated for Zika was also in Ticul (7.32 per 1,000 person-years). One case of co-infection of dengue and chikungunya was also identified. The majority of cases occurred from August to December 2015 which is historically considered the dengue season in Yucatan, Mexico. We explored the presence of clustering at the family level on this cohort but during this first year of follow-up in just one household were detected two dengue cases so the adjustment for clustering was not needed.

**Table 5:** Incidence rates (IR) per 1,000 person-years of arbovirus confirmed symptomatic infections in the cohort in Yucatan, Mexico by age, gender, and city.

	Person-years at risk (N)	Dengue	IR (95%CI)	Chikungunya	IR (95%CI)	Zika	IR (95%CI)
All participants	3430.9 (3400)	12	3.5 (1.9, 5.9)	30	8.6 (5.8, 12.3)	8	2.3 (0.9, 4.5)
Age groups (years)							
≤ 8	1001.4 (991)	3	2.9 (0.6, 8.8)	10	9.9 (4.8, 18.4)	4	3.99 (1.1, 10.2)
9–14	502.5 (484)	1	1.9 (0.03, 11.1)	4	7.9 (2.1, 20.4)	2	3.9 (0.5, 14.4)
15–19	145.3 (146)	1	6.88 (0.1, 38.3)	1	6.9 (0.1, 38.3)	0	0)
20–49	1490.1 (1456)	7	4.7 (1.9, 9.7)	11	7.4 (3.7, 13.2)	2	1.3 (0.2, 4.9)
≥ 50	291.5 (323)	0	0	4	13.7 (3.7, 35.1)	0	0
Gender							
Male	1511.7 (1531)	0	0	9	5.9 (5.8, 12.3)	3	1.9 (0.4, 5.8)
Female	1919.2 (1869)	12	6.3 (3.2,10.9)	21	10.9 (6.8, 16.7)	5	2.6 (0.8, 6.1)
City							
Merida	2164.3 (2021)	9	4.2 (1.9, 7.9)	18	8.3 (4.9, 13.1)	2	0.9 (0.1, 3.3)
Progreso	601.8 (641)	2	3.3 (0.4, 12.0)	5	8.3 (2.7,19.3)	1	1.7 (0.02, 2.3)
Ticul	682.9 (738)	1	1.5 (0.02, 8.2)	7	10.3 (4.1, 21.1)	5	7.3 (2.4, 17.1)

### Total dengue infections

For the analysis of dengue infections, we included only the participants who completed the first year of follow-up and contributed with a blood sample at the beginning of the study (starting in January 2015 through out 2015) and at the end of the first year of follow-up depending on the day of enrollment (up to October 2016). Of these 1,890 participants, 1,037 (54.8%) were from Merida, 379 (20.1%) were from Progreso and 474 (25.1%) were from Ticul. In total, these 1,890 participants contributed 2271.14 person-years and had 83 confirmed dengue infections, for an incidence rate of 36.55 infections per 1,000 person-years (95%CI 29.29, 45.07) (Table 6). From the total confirmed dengue infections, 14.5% (12/83) were confirmed using RT-PCR for dengue and the rest 85.5% (71/86) were confirmed by serology according to the protocol. The overall ratio of dengue infections to symptomatic cases was 8.22 dengue infections per dengue symptomatic case. The highest incidence of dengue was observed in the participants in the group of 15-19 years of age followed by the ≤ 8 year olds. High incidence rates were also observed in participants from Merida, females, and in the population of naïves (Table 6). The lowest rates of symptomatic cases were in the oldest age group(≥ 50 years-old) and the 9 to 14 years-old group with a rate of 6.66 cases per 100 infections (Additional tables by age and city can be found on the supplemental material S1 Table).

**Table 6:** Incidence rates (IR) per 1,000 person-years of dengue infections in the cohort in Yucatan, Mexico by age, gender, and city (N = 1,890).

	Person-years at risk (N)	Dengue infections	IR (95%CI)
All participants	2271(1890)	83	36.55 (29.3, 45.1)
Age (Years)			
≤ 8	692.84 (624)	35	50.5 (35.7, 69.5)
9–14	359.74 (305)	17	47.3 (28.5, 74.1)
15–19	92.91 (76)	9	96.9 (47.2, 177.8)
20–49	959.68 (757)	23	23.9 (15.6, 35.4)
≥ 50	166.11 (128)	5	30.1 (11.0, 66.7)
Gender			
Male	984.49 (882)	27	27.4 (18.4, 39.4)
Female	1286.79 (1008)	62	48.2 (37.3, 61.4)
City			
Merida	1248.14 (1032)	51	40.9 (30.7, 53.3)
Progreso	481.76 (401)	27	56.1 (37.7, 80.4)
Ticul	540.38 (457)	11	20.4 (10.7, 35.4)
Prior exposure to dengue			
Naïve	645.80 (542)	74	114.6 (90.6, 143)
Non-naïve	1625.20 (1348)	9	5.5 (2.7, 10.1)

### Primary dengue infections in the naïve population

Among the 829 participants who entered the cohort as dengue-naïve, 555 dengue-naïve participants completed the first annual of follow-up and provided a blood sample. These participants contributed 560.5 person-years of time. Over the first year of follow-up, 74 dengue-naïve participants experienced a primary dengue infection. The overall percent of seroconverting naïve individuals was 13.3%. The incidence rate of primary dengue infections was 132.0 infections per 1,000 person-years (95% CI: 104.4, 164.8). There were twice as many seroconverting naïve individuals in Progreso (14.8%) and Merida (14.2%), as compared to Ticul (7.7%) (Table 7).

**Table 7:** Seroconversion in the dengue-naïve participants at baseline in the first annual follow-up of the cohort in Yucatan, Mexico (n = 555).

	Merida (n = 302)	Progreso (n = 162)	Ticul (n = 91)	Total
Seroconversion in naïves				
Yes	43 (14.2%)	24 (14.8%)	7 (7.7%)	74 (13.3%)
No	259 (85.8%)	138 (85.2%)	84 (92.3%)	481 (86.7%)

### Incidence rate ratios of arbovirus confirmed symptomatic cases and dengue infections

For the IRR estimations, we assumed as exposed group the population that was naïve at baseline and the unexposed the non-naïves. The IRRs were estimated for confirmed dengue symptomatic cases, confirmed chikungunya symptomatic cases, confirmed Zika cases and overall dengue infections. The IRR for total dengue infections and symptomatic Zika cases were significant using the Logrank test (Table 8). More confirmed Zika cases were detected in dengue naïves and more confirmed chikungunya cases were detected in dengue non-naïves (Table 8).

**Table 8:** Incidence rate ratios for all arbovirus infections in the first annual follow-up of the cohort in Yucatan, Mexico.

Event	IRR (95%CI)	Logrank
Dengue confirmed cases	1.4 (0.47, 4.14)	p = 0.539
Dengue total infections	22.2 (11.13, 44.18)	p = 0.001
Chikungunya confirmed cases	0.5 (0.21,1.24)	p = 0.114
Zika confirmed cases	3.7 (1.06, 13.26)	p = 0.041
Any arboviral infection	0.9 (0.49, 1.61)	p = 0.625

Confirmed Zika cases were 3.7 times more likely in dengue naïve compared to non-naïve people. Also, for dengue total infections were 22.2 times more likely in dengue naïves compared to non-naïves. The incidence rate ratios by city and age can be found in the supplemental materials.

### Survival model for dengue infections

A Cox proportional hazard model was fitted for total dengue infections. The variables included in the model were: age, prior exposure to dengue, gender, city, household size of five or more people, and one or more infections in the same household. In the univariate analysis, age as a continuous variable was significant as a protective factor for dengue infections (HR = 0.98, 95%CI: 0.96, 0.99). There was no significant association between total dengue infections and overcrowding in the household but the remaining variables were significantly associated with having a dengue infection. The highest hazard ratios were estimated for baseline exposure to dengue (HR = 20.5, 95%CI: 10.30, 40.91) and having more people infected with dengue in the household (HR = 57.9, 95%CI:37.21, 90.08). Those two variables were included in the final model as potential confounders. In the final model, having a dengue infection during the first year of follow-up was significantly associated with female gender, living in Ticul or Progreso, and being dengue naïve at baseline. Age was not significantly associated with the outcome, as it was confounded by prior immunity to dengue that increases with age. (Table 9).

**Table 9:** Hazard ratios for total dengue infections in the first year of follow-up of the cohort in Yucatan, Mexico.

Variable	Hazard ratio	(95%CI)
Age		
≤ 8	Ref	Ref
9–14	1.06	0.59, 1.89
15–19	1.77	0.79, 3.96
20–49	1.27	0.72, 2.24
≥ 50	1.17	0.44,3.09
Gender		
Males	Ref	Ref
Females	1.65	1.04, 2.62
City		
Merida	Ref	Ref
Ticul	7.17	3.12, 16.49
Progreso	2.04	1.12, 3.76
Baseline status		
Dengue non-naïve	Ref	Ref
Dengue naïve	15.35	7.19, 33.08

## Discussion

To our knowledge this is the first prospective cohort study to describe the incidence rates of laboratory confirmed acute dengue and other arboviral infections in a population of healthy individuals in the state of Yucatan, Mexico. Our results confirm sustained dengue transmission and also the emergence of other arbovirus (chikungunya virus and Zika virus) since 2015 in the state of Yucatan. This cohort enrolled 767 families (3,400 participants) from three cities of the state of Yucatan, Mexico. The 3,400 participants contributed 3,480 person-years during the first year of follow-up. The estimated dengue baseline seroprevalence of the cohort was 70.28%. The city with the higher seroprevalence was Ticul (81.07%), followed by Merida and Progreso and these seroprevalences increased with age as expected for dengue endemic transmission settings. It is important to highlight that the seroprevalence in the age group from 9–14 years old in the three study settings were ≤ 70% and these findings could be relevant for the Mexican government to guide policies for dengue vaccine introduction.

Most of the dengue cohorts in Latin America and South East Asia are pediatric cohorts [29, 35, 37, 38, 45, 51–54]. Our cohort and the cohort from Iquitos, Peru have similar design, both have participants from all age groups and active surveillance was done at schools [55, 56]. Our cohort provides the opportunity to collect prospective data in all ages to understand better the full burden of dengue and other arboviruses that have emerged in the region.

The symptoms from the arboviral infections detected during the first year of follow-up were very similar which is why the clinical diagnosis might not be accurate. It is necessary to confirm the suspected cases in order to know which viruses are circulating in these endemic areas. The incidence rate of arboviral suspected cases was 58.2 per 1,000 person years. The dengue incidence rate for suspected cases was 21.86 per 1,000 person-years (95%CI 17.32, 27.25) and falls in the confidence interval of incidence rates estimated in the Nicaraguan dengue cohort [29, 57, 58].

The incidence rates of confirmed symptomatic Zika and confirmed chikungunya were 2.33 cases per 1,000 person-years and 8.74 cases per 1,000 person-years respectively. One case with co-infection of dengue and chikungunya was also detected as in some other endemic countries with co-circulation of multiple arbovirus [59]. The incidence rate of confirmed symptomatic dengue infections was 3.49 per 1,000 person-years. This proportion of confirmation and the incidence were lower than these estimates in other dengue cohorts [29, 35, 37, 38, 45, 51–54, 60].

One explanation is that during the first year of follow-up chikungunya virus was introduced in the state of Yucatan and these arboviruses that are transmitted by *Aedes aegypti* compete in the mosquito causing lower circulation of the other arbovirus in this case dengue. [61–64]

From the original cohort a total of 1,096 (32.2%) participants did not complete the activities of the first year of follow-up but 2,304 are still being followed from the original cohort. The mobility on the population in Yucatan was higher than we anticipated so assessing potential mobility will be important for the future of the cohort.

The analysis of the dengue infections was done in 1,890 participants who had a baseline and completed the first year of follow-up. The incidence rate for dengue infections was 36.55 infections per 1,000 person-years. This incidence rate is lower compared with other cohorts around the world but it concurs with the low transmission of dengue during this first-year of follow-up and the emergence of chikungunya in the state of Yucatan [29, 35, 37, 38, 45, 51–54, 60]. The highest incidence of dengue was observed in the participants in the group from 15-19 years of age followed by the ≤ 8 year-olds. The group from 15-19 years of age is just 4.65% of the cohort and only 25.64% of them were naïve at baseline. More participants from this age group have been enrolled in the last months so it is expected to have better denominators for the next years of follow-up.

Among 829 participants who were naïve at baseline, 555 (66.94%) completed the first year of follow-up. In the naïves, there were 74 confirmed dengue infections for an overall seroconversion rate of 13.33%. The incidence rate on primary infections was 114.59 per 1,000 person-years. This rate is very similar to estimates from other dengue prospective cohorts in endemic areas [29, 35].

Confirmed Zika cases were 3.7 times more likely in dengue naïve compared to non-naïve people. Also, dengue total infections were 22.2 times more likely in dengue naïves compared to non-naïves, this could be a reflection of inapparent infections that were missed in the population, and/or secondary infections that were not detected. The other incidence rate ratios estimated were not significant. This is a very interesting finding given the current interest in potential cross protection of dengue and Zika antibodies. It may be that recent, within two years, dengue infection may be protective against symptomatic Zika. Our results are consistent with recent publications available on this topic but do not prove the cross-protection and more research needs to be done [65, 66].

The hazard ratios estimated for dengue infections during the first year of follow-up were significant for females, participants living in Ticul or Progreso, one or more infections confirmed in the household, and being dengue naïve at baseline. In the final model after controlling for baseline dengue status age was not significantly associated with the outcome. To our knowledge, this is the first dengue cohort study that uses survival analysis as a tool to better understand the transmission dynamics of dengue and other arbovirus.

The limitations of this study include ascertainment of cases through enhanced passive surveillance. Thus, some dengue cases may not have been detected due to participants not seeking healthcare. Another limitation is that serum samples for cohort-wide serological testing are only available for participants once per year so it will be better to have more seroprevalence data points in time but logistically this is difficult to manage. We did not test the yearly samples for inapparent infections for CHIKV and ZIKV given that it was not planned on the original protocol and also due to the cross-reactivity of DENV and ZIKV with the currently available serological assays. Also we did not use paired samples in all the symptomatic arboviral cases; this decision was made with the advise of the arbovirus State Laboratory of Yucatan. This study relies on Inhibition ELISA to assess inapparent infections. The gold standard to assess DENV infection is the plaque reduction neutralization test (PRNT) but it is very labor intensive and expensive so testing all the participants was not feasible.

Over the last five decades, dengue has emerged as a major health problem in the tropical regions worldwide including Latin America and the Caribbean [2, 10, 22, 36]. More recently chikungunya and Zika virus emerged in the Americas causing significant epidemics in most of the countries infested with *Aedes aegypti* [67–70]. Mexico is considered one of the endemic dengue countries in the region and since 2015 with the emergence of chikungunya and Zika virus, the three arboviruses have been co-circulating in many areas of the country [71–73].

In summary, this study reported baseline seroprevalence, incidence of dengue infections, dengue cases and other arboviral cases in a community-based cohort from three settings in Yucatan, Mexico using analytic methods. This is the first report of the results from this prospective cohort that allowed to determine the incidence of arboviral infections and to estimate the true rate of disease, which is usually underestimated by passive surveillance. These data will be used to estimate disease burden. The incidence estimates will be useful for policy makers and to evaluate interventions like vector control strategies and vaccines. Future analysis of household transmission, cluster analysis and effectiveness of interventions are planned.

## Supporting information

S1 Checklist STROBE Checklist for cohort studies. (PDF)

S1 Table Incidence rate ratios by city. (PDF)

S2 Table Incidence rate ratios by age. (PDF)
