# Supplementary material for: Epidemiology of dengue and other arboviruses in a cohort of school children and their families in Yucatan, Mexico: Baseline and first year follow-up
Source: PLoS Negl Trop Dis. 2018 Nov 21;12(11):e0006847. doi: 10.1371/journal.pntd.0006847 (PMC6248893; doi:10.1371/journal.pntd.0006847)
Supplement: S1 Checklist — (PDF) [file pntd.0006847.s001.pdf]

STROBE Statement—Checklist of items that should be included in reports of ***cohort studies***

	Item No	Recommendation
**Title and abstract**	1	(*a*) Indicate the study's design with a commonly used term in the title or the abstract [**Abstract, paragraph 1**]
		(*b*) Provide in the abstract an informative and balanced summary of what was done and what was found. [**Abstract, page 1-2**]
**Introduction**		
Background/rationale	2	Explain the scientific background and rationale for the investigation being reported [**Introduction, page 2-3**]
Objectives	3	State specific objectives, including any prespecified hypotheses [P**age 3 lines 46-50**]
**Methods**		
Study design	4	Present key elements of study design early in the paper [**Methods, page 3 lines 71-72**]
Setting	5	Describe the setting, locations, and relevant dates, including periods of recruitment, exposure, follow-up, and data collection. [**Methods, page 3 lines 53-68**]
Participants	6	(*a*) Give the eligibility criteria, and the sources and methods of selection of participants. Describe methods of follow-up [**Methods, page 4-5 lines 87-144**]
		(*b*) For matched studies, give matching criteria and number of exposed and unexposed [**NA**]
Variables	7	Clearly define all outcomes, exposures, predictors, potential confounders, and effect modifiers. Give diagnostic criteria, if applicable [**Methods, page 5-6 lines 121-200**]
Data sources/ measurement	8*	For each variable of interest, give sources of data and details of methods of assessment (measurement). Describe comparability of assessment methods if there is more than one group [**Methods, page 5-6 lines 121-200**]
Bias	9	Describe any efforts to address potential sources of bias [**Methods, page 7 lines 212-234**]
Study size	10	Explain how the study size was arrived at [**Methods, page 4 lines 82-85**]
Quantitative variables	11	Explain how quantitative variables were handled in the analyses. If applicable, describe which groupings were chosen and why[**Methods, page 7 lines 215-221**]
Statistical methods	12	(*a*) Describe all statistical methods, including those used to control for confounding [**Methods, page 7 lines 215-237**]
		(*b*) Describe any methods used to examine subgroups and interactions [**Methods, page 7 lines 235-236**]
		(*c*) Explain how missing data were addressed [**NA**]
		(*d*) If applicable, explain how loss to follow-up was addressed [**Methods, page 7 lines 218-221**]
		(*e*) Describe any sensitivity analyses [**NA**]
**Results**		
Participants	13*	(a) Report numbers of individuals at each stage of study—eg numbers potentially eligible, examined for eligibility, confirmed eligible, included in the study, completing follow-up, and analysed [**Results, page 7 lines 240-251**]
		(b) Give reasons for non-participation at each stage [**Results, page 7 lines 240-251**]
		(c) Consider use of a flow diagram [**Results, page 7 , Fig 1**]
Descriptive data	14*	(a) Give characteristics of study participants (eg demographic, clinical, social) and information on exposures and potential confounders [**Results, page 8 Table 1**]
		(b) Indicate number of participants with missing data for each variable of interest [**NA**]
		(c) Summarise follow-up time (eg, average and total amount) [**Results, page 7 lines 245-246**]
Outcome data	15*	Report numbers of outcome events or summary measures over time [**Results, page 9-12]**

Main results	16	(*a*) Give unadjusted estimates and, if applicable, confounder-adjusted estimates and their precision (eg, 95% confidence interval). Make clear which confounders were adjusted for and why they were included [**Results, page 10 table 5, page 12 lines 341-355**]
		(*b*) Report category boundaries when continuous variables were categorized [**Results, all tables]**
		(*c*) If relevant, consider translating estimates of relative risk into absolute risk for a meaningful time period [**Results, page 12**]
Other analyses	17	Report other analyses done—eg analyses of subgroups and interactions, and sensitivity analyses
**Discussion**		
Key results	18	Summarise key results with reference to study objectives [**Discussion, page 12, lines 357-366]**
Limitations	19	Discuss limitations of the study, taking into account sources of potential bias or imprecision. Discuss both direction and magnitude of any potential bias [**Discussion, page 14, lines 428-438]**
Interpretation	20	Give a cautious overall interpretation of results considering objectives, limitations, multiplicity of analyses, results from similar studies, and other relevant evidence [**Discussion, page 13-14]**
Generalisability	21	Discuss the generalisability (external validity) of the study results [**Discussion, page 14-15, lines 439-454]**
**Other information**		
Funding	22	Give the source of funding and the role of the funders for the present study and, if applicable, for the original study on which the present article is based [**Funding, page 15**]

*Give information separately for exposed and unexposed groups.

Note: An Explanation and Elaboration article discusses each checklist item and gives methodological background and published examples of transparent reporting. The STROBE checklist is best used in conjunction with this article (freely available on the Web sites of PLoS Medicine at http://www.plosmedicine.org/, Annals of Internal Medicine at http://www.annals.org/, and Epidemiology at http://www.epidem.com/). Information on the STROBE Initiative is available at http://www.strobe-statement.org.
