# Supplementary material for: Epidemiology of dengue and other arboviruses in a cohort of school children and their families in Yucatan, Mexico: Baseline and first year follow-up
Source: PLoS Negl Trop Dis. 2018 Nov 21;12(11):e0006847. doi: 10.1371/journal.pntd.0006847 (PMC6248893; doi:10.1371/journal.pntd.0006847)
Supplement: S1 Table — (PDF) [file pntd.0006847.s002.pdf]

Supplemental material

September 27, 2018

Epidemiology of Dengue and Other Arboviruses in a Cohort of School Children and Their Families in Yucatan, Mexico: Baseline and First Year Follow-up.

Table 1

Table 1: **Incidence rate ratios by city for all arbovirus infections in the first annual follow-up of the cohort in Yucatan, Mexico.**

Event	IRR (95%CI)
**Merida**	
Dengue confirmed cases	1.99 (1.10, 3.59)
Dengue total infections	22.9 (12.71, 41.31)
Chikungunya confirmed cases	0.24 (0.13, 0.43)
Zika confirmed cases	0
Any arboviral infections	4.88 (2.71, 8.81)
**Progreso**	
Dengue confirmed cases	0
Dengue total infections	17.93 (9.94, 32.32)
Chikungunya confirmed cases	2.15 (1.19, 3.88)
Zika confirmed cases	0
Any arboviral infections	10.76 (5.96, 19.39)
**Ticul**	
Dengue confirmed cases	2.36 (1.30, 4.26)
Dengue total infections	21.26 (11.79, 38.33)
Chikungunya confirmed cases	0.79 (0.44,1.42)
Zika confirmed cases	0.62 (0.35, 1.12)
Any arboviral infections	4.32 (2.40, 7.81)
