# Supplementary material for: Epidemiology of dengue and other arboviruses in a cohort of school children and their families in Yucatan, Mexico: Baseline and first year follow-up
Source: PLoS Negl Trop Dis. 2018 Nov 21;12(11):e0006847. doi: 10.1371/journal.pntd.0006847 (PMC6248893; doi:10.1371/journal.pntd.0006847)
Supplement: S2 Table — (PDF) [file pntd.0006847.s003.pdf]

# Supplemental tables

September 27, 2018

**Epidemiology of Dengue and Other Arboviruses in a Cohort of School Children and Their Families in Yucatan, Mexico: Baseline and First Year Follow-up.**

## Table 2

Table 1: **Incidence rate ratios by age for all arbovirus infections in the first annual follow-up of the cohort in Yucatan, Mexico.**

| Event | IRR (95%CI) |
|---|---|
| $\leq 8$ **year olds** | |
| Dengue confirmed cases | 2.44 (0.82, 7.29) |
| Dengue total infections | 27.70 (15.36, 49.94) |
| Chikungunya confirmed cases | 0.31 (0.12, 0.73) |
| Zika confirmed cases | 4.07 (1.15, 14.43) |
| Any arboviral infections | 3.42 (1.90, 6.17) |
| **9 -14 year olds** | |
| Dengue confirmed cases | 0 |
| Dengue total infections | 23.35 (12.95, 42.11) |
| Chikungunya confirmed cases | 1.37 (0.57, 3.31) |
| Zika confirmed cases | 1.37 (0.38, 4.87) |
| Any arboviral infections | 9.16 (5.08, 16.51) |
| **15-19 year olds** | |
| Dengue confirmed cases | 0 |
| Dengue total infections | 18.85 (10.45, 34.01) |
| Chikungunya confirmed cases | 0 |
| Zika confirmed cases | 0 |
| Any arboviral infections | 9.43 (5.23, 17.02) |
| **20-49 year olds** | |
| Dengue confirmed cases | 0 |
| Dengue total infections | 23.85 (13.22, 43.00) |
| Chikungunya confirmed cases | 0.75 (0.31, 1.79) |
| Zika confirmed cases | 0 |
| Any arboviral infections | 7.71 (3.20, 18.57) |
| $\geq 50$ **year olds** | |
| Dengue confirmed cases | 0 |
| Dengue total infections | 49.46 (27.43, 89.19) |
| Chikungunya confirmed cases | 0 |
| Zika confirmed cases | 0 |
| Any arboviral infections | 98.93 (54.86, 178.38) |
